# The Influence of Body Position on the Resting Motor Threshold of Posterior Root-Muscle Reflexes Evoked via Transcutaneous Spinal Cord Stimulation

**DOI:** 10.3390/jcm13175008

**Published:** 2024-08-23

**Authors:** Barry T. Gorman, Conor Gill, Mark Etzelmueller, Clodagh O’Keeffe, Richard B. Reilly, Neil Fleming

**Affiliations:** 1 Discipline of Anatomy, School of Medicine, Trinity College Dublin, D02 R590 Dublin, Ireland; neil.fleming@tcd.ie; 2 School of Engineering, Trinity College Dublin, D02 R590 Dublin, Irelandokeeffcl@tcd.ie (C.O.); reillyri@tcd.ie (R.B.R.); 3 Discipline of Gerontology, School of Medicine, Trinity College Dublin, D02 R590 Dublin, Ireland; 4 Trinity Centre for Biomedical Engineering, Trinity College Dublin, D02 R590 Dublin, Ireland

**Keywords:** tSCS, transcutaneous, PRMR, posterior root-muscle reflex, neuromodulation, lower limb, spinal cord injury, SCI, spine alignment, spine anatomy

## Abstract

**Background:** Thoracolumbar transcutaneous spinal cord stimulation (tSCS) non-invasively evokes posterior root-muscle reflexes (PRMR) with the aim of neuromodulating sensorimotor function following spinal cord injury. Research is still in its infancy regarding the effect of body position on the nature of these spinally evoked responses. Therefore, the aim of this study was to investigate the influence of body position on the nature of PRMR responses during tSCS. **Methods:** A total of 11 (6M, 5F) participants completed a full PRMR recruitment curve from 10 ma up to 120 ma (10 ma increments) at the T11/12 intervertebral space using a singular 3.2 cm diameter cathode. At each intensity, three paired pulses (50 ms inter-pulse interval), followed by three singular pulses with a six-second delay were applied in each body position (supine, supine 90-90, sitting and standing) in a randomised order. The PRMR responses in lower limb muscles were recorded using wireless electromyographic sensors placed on the *Soleus*, *Tibialis Anterior*, *Rectus Femoris* and *Bicep Femoris* long head. A two-way (body position × muscle) repeated measures analysis of variance was used to investigate the effect of body position on PRMR-evoked responses. **Results:** There was a significant main effect of body position on PRMR resting motor threshold (RMT) (*p* < 0.001), first response peak-to-peak amplitude (*p* = 0.003) and percentage post-activation depression (%PAD) (*p* = 0.012). Sitting had significantly higher RMT and significantly lower first response peak-to-peak amplitudes compared to all other positions, but significant differences in %PAD were only detectible between supine and standing. **Conclusions:** Body position influences the nature of PRMR-evoked responses during tSCS.

## 1. Introduction

Millions of people around the world are affected by spinal cord injuries (SCI), most often as a result of high-force traumatic incidents involving motor collisions or sporting accidents [1]. Such injuries not only affect a person’s physical and mental health but profoundly impact their ability to live independent lives [2,3,4]. Thus, novel bioengineering approaches towards improved activities of daily living for those with SCI continue to be developed and tested. These solutions include restoration (functional electrical stimulation) [5] and replacement approaches (brain–machine interfaces) [6] along with various neuromodulation techniques [7,8]. The latter is broadly defined as any approach that aims at inducing plasticity of the central nervous system (CNS) either through stimulation of sensory afferents or through the use of feedback provided by efferent signals [9]. 

Transcutaneous spinal cord stimulation (tSCS) is one such neuromodulation approach that has become increasingly popular over the past decade. This approach involves the reflex activation of spinal networks below the site of injury, using trains of electrical current delivered via electrodes placed on the skin overlying the lumbo–thoracic enlargement [10,11,12]. Sayenko et al. [13] previously demonstrated that tSCS activates distinct proximal or distal motor pools via sensory fibres in dorsal roots of the spinal cord in accordance with the anatomical arrangement along the rosto–caudal axis. Taking this into account, activating motor pools through non-invasive dorsal root or interneuron stimulation in those with SCI could potentially lower their activation threshold as a result of residual supraspinal input [14]. The ability to activate various spinal cord neural networks non-invasively has opened the therapeutic window for the treatment of consequent motor dysfunction following SCI.

tSCS is thought to directly stimulate the medium to large-diameter sensory afferents located in the dorsal rootlets [11,15]. The resultant reflex responses are recorded via electromyography (EMG) in the downstream muscles and are termed posterior-root-muscle reflexes (PRMR). These PRMRs are thought to share their short latency with the Hoffmann reflex [16]. Paired pulse stimuli with short inter-pulse intervals (IPI) of 30–50 ms are typically employed to confirm the trans-synaptic reflex origin of PRMRs [17]. The short IPI means that insufficient neurotransmitters can be released pre-synaptically from the sensory afferents during the second stimulation, resulting in the attenuation or complete eradication of the downstream response (PRMR2). This phenomenon is known as post-activation depression (PAD) and it specifically confirms the reflex nature of tSCS [11,15]. By comparing the conditioning response (PRMR1) with the test motor response (PRMR2), PAD can be quantified. Significant PAD has previously been recorded in the lower limb muscles during tSCS of the lumbo–thoracic region under controlled conditions [11,15,18].

While the therapeutic benefits of tSCS in SCI rehabilitation are well documented [19], the reliable application appears crucial to allow selective activation of the sensory afferents [20]. However, recent reviews have highlighted the considerable variance in the methods of lumbo–thoracic tSCS being employed [17,19], with a lack of agreement on the “optimal” body position for stimulation. Discrepancies in body position and cathode location could preferentially activate motor fibres rather than sensory fibres and therefore limit any neuromodulation. Therefore, identifying this “optimal” body position is important as it could be utilised as the body position for therapeutic tSCS intervention in those with a SCI. While tSCS has been utilised in various body positions [11,21,22,23,24,25], only one study has directly compared the effect of different body positions on the resultant evoked responses [26]. This study concluded that body position significantly impacted the level of trans-synaptic reflex activation observed. More specifically, stimulation when participants were lying prone resulted in significantly lower levels of PAD and higher resting motor thresholds compared to lying supine or standing. This suggests that the target sensory afferents within the dorsal rootlets may not be stimulated as effectively in a prone position, therefore reducing the reflex nature of the tSCS.

Binder et al. [20] subsequently compared the effect of spinal flexion angle across body positions in order to evaluate if body position differences, which were previously observed (by Danner et al. [26]), were mediated by changes in the vertebral alignment. While the authors did not statistically compare body positions, they did show that the PRMR responses were significantly altered with changes in the flexion angle of the lumbar spine. Specifically, the authors reported lower levels of PAD with a flexed spine compared to a neutral and extended spine. This suggested that moving the spine into a more flexed position results in the co-activation of both sensory and motor fibres in their respective dorsal and ventral rootlets and changes in the level of PAD may be anatomical or biophysical in nature rather than centrally mediated.

While Binder et al. [20] must be applauded for the direct comparison of H-reflex responses to tSCS, matching the stimulation intensities between the two approaches in some ways limited the findings, as no information on the full intensity–response curve was provided, but rather, data at one particular intensity only. Nor do we know the effect that body position had on RMT, which is arguably the most important descriptor of tSCS stimulation characteristics, particularly for therapeutic dosage of tSCS (the main reason this work is being conducted at all). Taken together, Danner et al. [26] and Binder et al. [20] have concluded that tSCS applied while lying prone or with a flexed spine alignment likely reduces trans-synaptic reflex activation of the lower limb muscles and instead results in more preferential stimulation of motor fibres in the ventral rootlets. Yet, neither study statistically compared sitting with the other body positions, a limitation that this current study aims to answer. Therefore, the aim of this exploratory study was to compare the influence of key body position associated with the sit-to-stand transition on resting motor threshold (RMT) and trans-synaptic origin (PAD) of evoked responses in muscles of the lower limb via lumbo–thoracic tSCS.

## 2. Materials and Methods

### 2.1. Participants

An a priori sample size calculation was conducted using G*Power (version 3.1.9.6) with a power of 0.95, effect size (N^2^_p_) of 0.707 for body position (Danner et al. [26]) and alpha level set at 0.05, and determined that a sample size of n = 8 was required. Eleven neurologically intact individuals subsequently participated in this study (6M, 5F. Mean age: 27 ± 6 years, height: 171.6 ± 10.7 cm, weight: 69.8 ± 12.9 kg). Participants were excluded if any of the following criteria were met (adapted from Rossi et al. [27]): injury to the spine or any neurological condition affecting control of motor function, postural abnormalities, cognitive impairment, previous surgery to the spine or abdomen, pregnancy, history of epilepsy, history of fainting spells or syncope, pacemaker or implanted metal in the body, skin condition over stimulation sites or obesity (BMI > 30 kg/m^2^). Obese participants were excluded as even experienced individuals find it difficult to identify bony landmarks [28]. Ethical approval was granted by a Faculty Level II Research Ethics Committee and all protocols were undertaken in accordance with the Declaration of Helsinki guidelines. Each participant also underwent medical screening and signed an informed consent form prior to their involvement in the study. 

### 2.2. Surface Electromyography

Wireless electromyographic (EMG) sensors (Delsys Trigno, Delsys Inc., Natwick, MA, USA) were placed on four lower limb muscles: *Soleus* (SOL), *Tibialis Anterior* (TA), *Rectus Femoris* (RF) and *Bicep Femoris* long head (BF_LH_). An artifact electrode was also placed on the abdomen, superior to the umbilicus. These sensors were placed on the skin, running parallel to the respective muscle fibres in accordance with SENIAM guidelines published by Hermens et al. [29]. Compound muscle action potentials were subsequently recorded at 2000 Hz and wirelessly transmitted to a laptop (Dell Alienware m15 R2, with an Intel ^®^ Core™ i5-9750H ninth generation processor and 16 GB RAM) via Delsys Trigno biofeedback EMG system. EMG signals were then processed in MATLAB (R2020a 9.8.0.1323502, MathWorks, Natwick, MA, USA) in order to quantify the peak-to-peak amplitude of each evoked response. 

### 2.3. Protocol

Two 5 × 10 cm rectangular electrodes (Axelgaard Manufacturing Co., Ltd., Fallbrook, CA, USA) were placed 2 cm on either side of the umbilicus. One circular cathode 3.2 cm in diameter (Axelgaard Manufacturing Co., Ltd., Fallbrook, CA, USA) was placed in the T11/12 intervertebral space. In order to identify the T11/12 intervertebral space, the participant lay in the prone position and the investigator palpated the inferior angle of the scapula as it aligned with T7. From there, the investigator counted down to T11/12. A Mediroyal Lumbar Support Belt was then placed on the participant according to their height and the shoulder and chest straps were fully tightened. This support belt had an integrated rigid spinal insert made with dural aluminum and provided rigid support from the upper thoracic to thoracolumbar regions. It was applied as a means of controlling flexion and extension movements of the spine throughout the different body positions, as spinal alignment has been shown to significantly affect the nature of evoked responses during tSCS [20]. An adjustable ankle orthosis (Atlas Adjustable Night Splint) was also employed to maintain SOL muscle length throughout the data recordings. 

### 2.4. Body Positions

**Figure 1:**
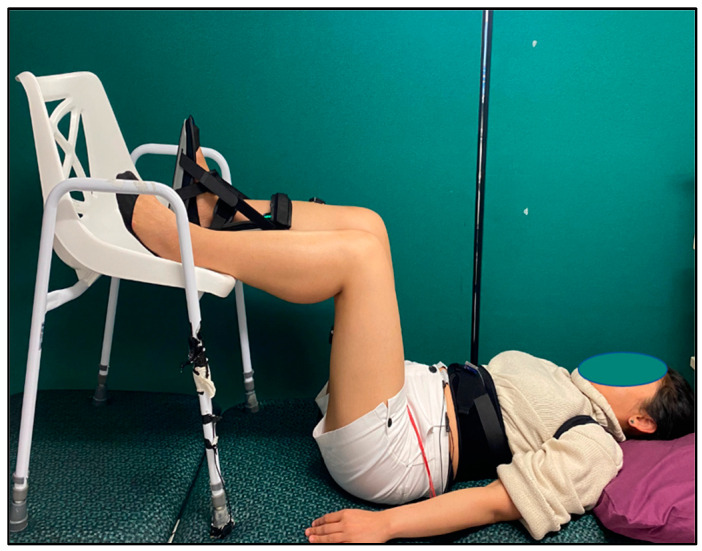
Supine 90-90 body position.

Each participant underwent a full PRMR recruitment curve using tSCS in supine, standing, sitting and supine 90-90 body positions, with responses being recorded in SOL, TA, RF and BF_LH_. The order of body position was randomised prior to the start of the testing. During supine and standing body positions, participants’ arms were relaxed and rested by their sides. During standing, participants stood upright against a wall, using it as a reference. The sitting position involved the participants sitting in a relaxed fashion, with their elbows flexed to 90°, hands on their thighs and their hips, knees and ankles maintained at 90° angles. During the supine 90-90 position, participants lay in the supine position with their hips and knees at 90° angles, arms by their sides and their lower legs resting on a chair (Figure 1). 

### 2.5. Stimulation Procedure

A Digitimer DS8R bipolar constant current stimulator was used to deliver the stimulations. The stimulation procedure involved three paired pulses at 50 ms IPI [11] with a 0.2 Hz frequency, followed by three single pulses applied with a six-second delay between consecutive pulses, similar to Danner et al. [26]. The stimulus intensity began at 10 mA and increased in 10 mA increments to 120 mA. Stimulations were ceased when maximal intensity was reached or at the participant’s request. This process was repeated for each body position. 

### 2.6. Data Analysis

**Figure 2:**
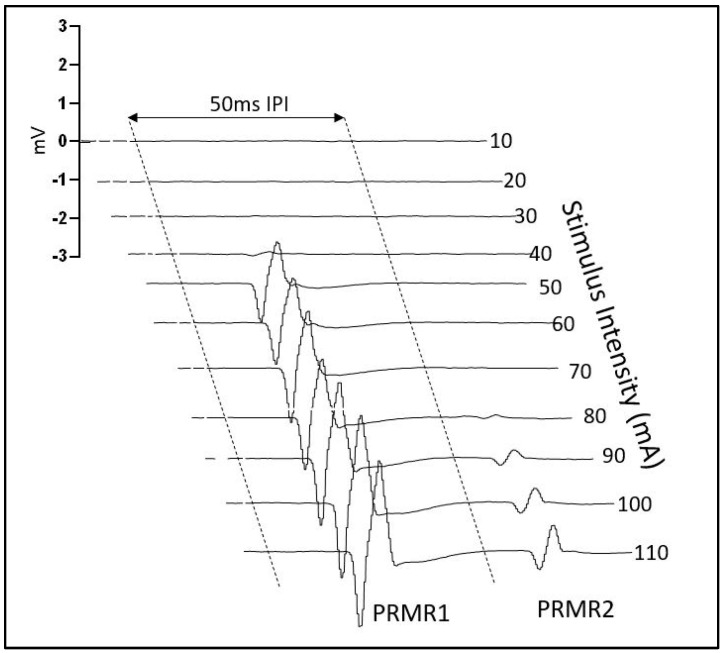
Exemplary trace of Soleus PRMRs at paired pulses from 10 mA to 110 mA.

The resulting surface EMG data collected from PRMR recruitment was recorded in each of the four muscles. The EMG sensors specifically contained a four-bar formation with 10 mm inter-electrode distance and a bandwidth of 20–450 Hz. The compound muscle action potentials were subsequently recorded at 2000 Hz and wirelessly transmitted to the Dell Alienware laptop via the Delsys Tringo biofeedback EMG system. The EMG signals were then processed in MATLAB in order to quantify the mean peak-to-peak amplitude of PRMR1 (first response) and PRMR2 (second response) to the single and double pulses at each intensity. The window of interest for PRMR1 responses was between 10 ms and 45 ms and for PRMR2 responses was between 60 ms and 90 ms (see Figure 2). PAD was then calculated as a percentage (%PAD) using the formula below:

$$\left(1-\left(\frac{PRMR2\;amplitude}{PRMR1\;amplitude}\right)\right)\times 100$$

**Figure 3:**
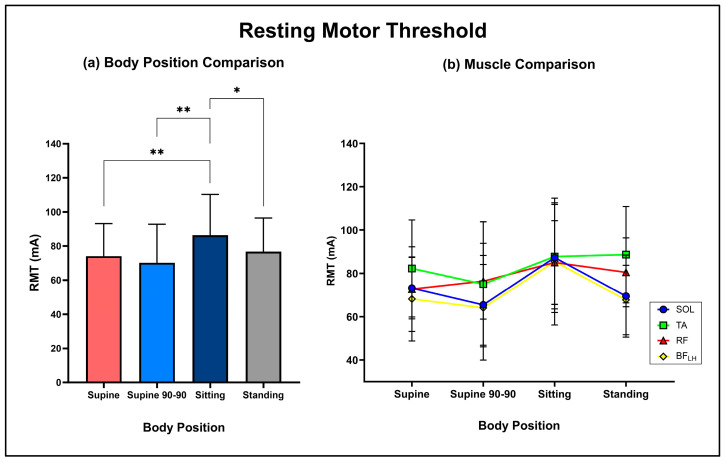
(**a**) Comparison of average PRMR resting motor threshold (defined as the lowest current required to elicit a peak-to-peak amplitude response greater than 50 uV in PRMR1) between supine, supine 90-90, sitting and standing (*: *p* = 0.03, **: *p* < 0.001). (**b**) Average PRMR RMTs for each muscle group between body positions (SOL: Soleus, TA: Tibialis Anterior, RF: Rectus Femoris, BF_LH_: Bicep Femoris long head).

**Figure 4:**
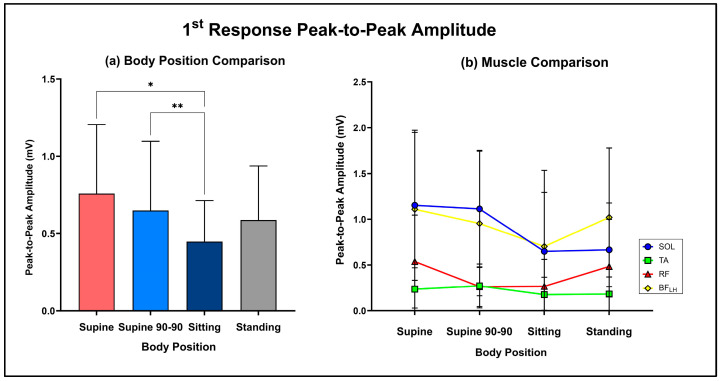
(**a**) Comparison of average PRMR1 amplitudes between supine, supine 90-90, sitting and standing (*: *p* = 0.012, **: *p* = 0.002). (**b**) Average PRMR1 response amplitudes of SOL, TA, RF and BF_LH_ during each of the body positions.

**Figure 5:**
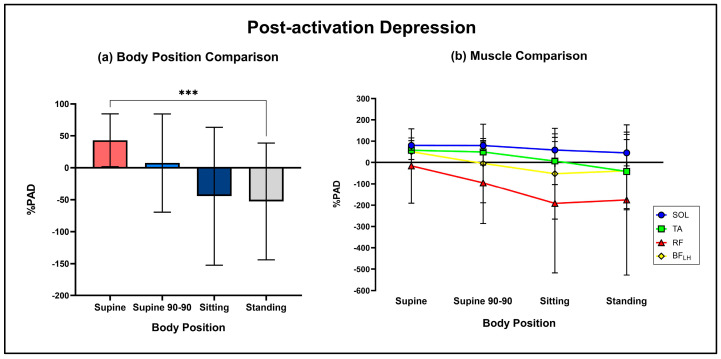
(**a**) Comparison of average %PAD between supine, supine 90-90, sitting and standing (***: *p* < 0.001). (**b**) Average %PAD of SOL, TA, RF and BF_LH_ during each of the body positions.

RMTs were measured during each body position and processed solely in MATLAB. RMT was defined as the lowest current required to elicit a peak-to-peak amplitude response greater than 50 uV in PRMR1. RMT, PRMR1 response amplitude and %PAD are herein denoted as the average response of all muscles (including Figure 3a, Figure 4a and Figure 5a). Recruitment curves were also generated by calculating average peak-to-peak amplitudes for PRMR1 and PRMR2 at each current intensity from 10 mA to 120 mA for each muscle and body position. 

### 2.7. Statistical Analysis

All statistical analyses were performed using GraphPad Prism (version 9.4.0.6 GraphPad Software, San Diego, CA, USA). Mean ± standard deviation was used to denote the descriptive statistics. Parametric tests were utilised as all data were normally distributed. A two-way (body position × muscle) repeated measures ANOVA with post hoc analysis (Bonferroni correction) was used to determine the effect of body position on RMT, first response (PRMR1) peak-to-peak amplitude and %PAD in all four muscles (SOL, TA, RF and BF_LH_). PRMR1 and %PAD were both compared at maximal stimulation intensity (120 mA: n = 7, 110 mA: n = 1, 100 mA: n = 1, 90 mA: n = 1, 80 mA, n = 1). Statistical significance was set at *p* < 0.05. 

## 3. Results

### 3.1. Body Positional Effect on PRMR RMT

The two-way mixed effects ANOVA demonstrated a significant main effect of body position (supine: 74 ± 19 mA, supine 90-90: 70 ± 23 mA, sitting: 86 ± 24 mA, standing: 77 ± 20 mA; F (2,95) = 12.7, *p* < 0.001) (Figure 3a), with Bonferroni-adjusted post hoc analysis revealing sitting had a significantly higher RMT compared to supine (mean diff: 12 mA; *p* < 0.001), supine 90-90 (mean diff: 16 mA; *p* < 0.001) and standing (mean diff: 10 mA; *p* = 0.03). There was no significant main effect of the muscle group (SOL: 74 ± 9 mA, TA: 83 ± 6 mA, RF: 79 ± 5 mA, BF_LH_: 71 ± 10 mA; F (3,40) = 0.9, *p* = 0.47) and no significant interaction effect (F (9,119) = 1.18, *p* = 0.32). Figure 3b displays the average PRMR1 RMT for each muscle group within each body position.

### 3.2. Body Positional Effect on PRMR1 Amplitude

Two-way repeated measure ANOVA demonstrated a significant main effect of body position (supine: 0.76 ± 0.45 mV, supine 90-90: 0.70 ± 0.45 mV, sitting: 0.45 ± 0.27 mV, standing: 0.59 ± 0.35 mV; F (2,76) = 6.5, *p* = 0.003, N^2^_p_ = 0.03). Bonferroni-adjusted post hoc analysis revealed that PRMR1 responses were significantly lower in sitting compared to both supine (mean diff: 0.32 mV; *p* = 0.012) and supine 90-90 (mean diff: 0.20 mV; *p* = 0.002) body positions (Figure 4a). 

The effect of the muscle group was also significant (SOL: 0.90 ± 0.28 mV, TA: 0.22 ± 0.05 mV, RF: 0.39 ± 0.14 mV, BF_LH_: 0.95 ± 0.18 mV; F (3,40) = 6.1, *p* = 0.002, N^2^_p_ = 0.23). SOL had significantly greater first responses than TA (mean diff: 0.67 mV; *p* < 0.001) and RF (mean diff: 0.57 mV; *p* < 0.001). Similarly, BF_LH_ had significantly greater first responses compared to TA (mean diff: 0.72 mV; *p* < 0.001) and RF (mean diff: 0.56 mV; *p* < 0.001). There was also a significant interaction effect (F (9,120) = 1.985, *p* = 0.047, N^2^_p_ = 0.04), highlighting that a muscle’s PRMR1 response amplitude varied depending on body position. The average PRMR1 responses of each muscle group across each body position can be seen in Figure 4b. 

### 3.3. Body Positional Effect on %PAD

The two-way repeated measure ANOVA demonstrated a significant main effect of body position (supine: 42.99 ± 41.42%, supine 90-90: 7.41 ± 76.79%, sitting: −44.45 ± 107.9%, standing: −52.65 ± 91.34%; F (2,82) = 4.7, *p* = 0.012, N^2^_p_ = 0.05) on %PAD. Bonferroni-adjusted post hoc analysis revealed that %PAD was significantly greater in standing compared to supine (mean diff: 95.64%; *p* < 0.001) (Figure 5a) body positions. 

There was also a significant main effect of the muscle on %PAD (SOL: 65.99 ± 41.13%, TA: 18.13 ± 114.6%, RF: −119.5 ± 272.4%, BF_LH_: −11.27 ± 25.29%; F (3,40) = 4.6, *p* = 0.007, N^2^_p_ = 0.14). Across body positions, SOL had the significantly greatest inhibitory response of all muscles (all *p* < 0.05). Bonferroni-adjusted post hoc analysis revealed a mean difference of 47.9% (*p* = 0.03), 185.5% (*p* < 0.001) and 77.3% (*p* < 0.001) between SOL and TA, RF and BF_LH_, respectively. It was also revealed that the %PAD observed in TA was significantly different from RF (mean diff: 137.7%; *p* = 0.008). There was no significant interaction effect (F (9,120) = 4.7, *p* = 0.84). Figure 5b displays the average %PAD for each muscle group between the different body positions.

**Figure 6:**
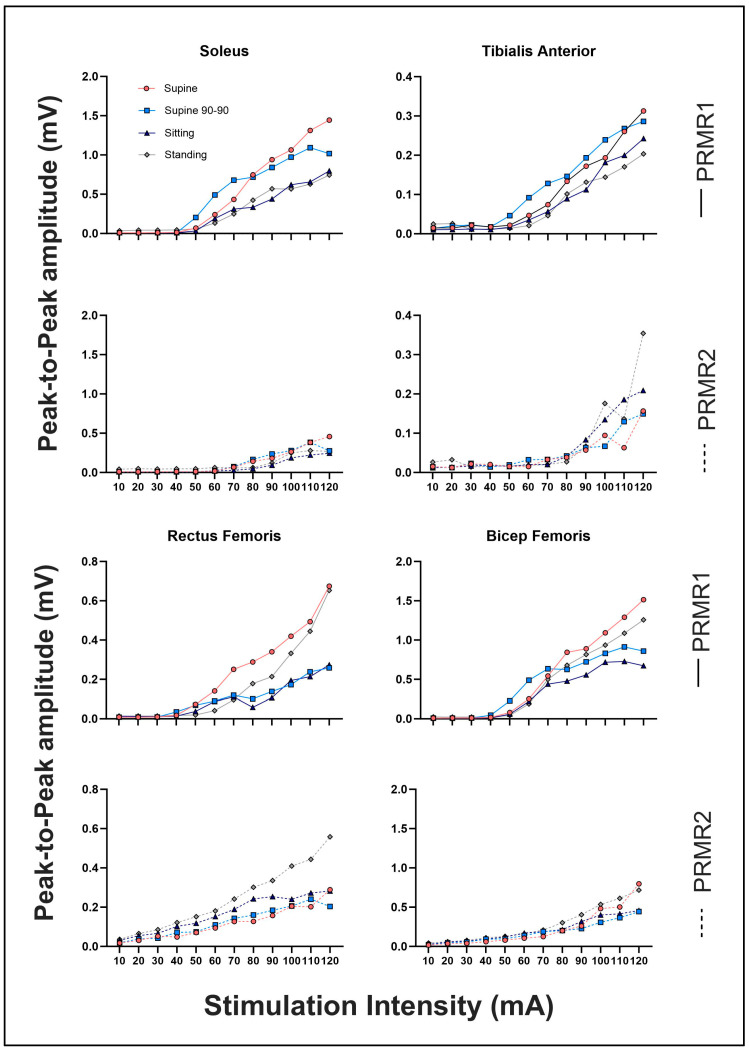
Average PRMR1 and PRMR2 recruitment curves for SOL, TA, RF and BF_LH_ during supine, supine 90-90, sitting and standing (full line indicates PRMR1. Dashed line indicates PRMR2).

The corresponding recruitment curves of the average PRMR1 and PRMR2 responses for SOL, TA, RF and BF_LH_ during each body position are depicted in Figure 6. 

## 4. Discussion

We found that body position affects RMT and the nature of evoked PRMR responses during tSCS. Particularly, sitting resulted in higher RMTs and lower peak-to-peak first response (PRMR1) amplitudes, yet statistical differences in %PAD were only observed between supine and standing body positions. 

Various inconsistencies have been identified regarding the optimal body position for the application of tSCS [17,19]. Body positional comparisons have been made in two studies [20,26], yet our study is the first to statistically compare the sitting position. In contrast to both Danner et al. [26] and Binder et al. [20], a spinal brace was utilised in this study, controlling for flexion and extension movements of the spine throughout all body positions. The spinal brace was used as Binder et al. [20] previously reported significantly reduced first responses and increased co-activation of motor fibres during maximal spinal flexion across supine, standing, sitting and lateral recumbent body positions. The use of this brace therefore eliminated the effect spinal flexion and extension movements may have had on observed responses and instead allowed for a more detailed examination of the central effects of each body position. Furthermore, a prone body position in comparison to supine and standing also demonstrated reduced first responses and increased stimulation of motor efferents, at an almost one-to-one ratio [26]. Importantly, unlike Binder et al. [20], Danner et al. [26] also investigated RMT and again found that the prone position had the highest RMT compared to supine and standing (*p* < 0.001). Both studies theorise that biophysical rather than central effects are responsible for these response differences between body positions and/or spinal alignments. Danner et al. [26] stated that due to the spinal cord migrating anteriorly as one moves from a supine to prone position [30], the prone body position preferentially influences the trajectory of the root fibres and their subsequent “hot-spot” threshold. Meanwhile, Binder et al. [20] suggested that rotating and/or translating vertebrae as one flexes and extends the spine alters the volumes engrossed by the vertebral canal’s soft tissues and fluids, as well as the size of the intervertebral foramina, and therefore alters the current flowing through. While the results of our study do not necessarily contradict this previous work, they do call into question the theory that biophysical and/or anatomical variance is the sole cause of evoked response differences between body positions. Specifically, the results of our study suggest that supine and supine 90-90 body positions primarily favour sensory fibre stimulation due to the significantly larger first responses (PRMR1) compared to sitting, along with the low second response amplitudes. Whilst sitting, RMTs are significantly higher than all other body positions and PRMR1 response amplitudes are significantly lower than supine and supine 90-90. 

The preferential activation of afferent fibres in the supine position is in agreement with Danner et al. [26]. However, in contrast to Danner et al. [26], who observed significant suppression of the second response during standing, instead, standing resulted in an excitatory response in our study. This result may have been largely swayed by the excitatory nature of RF throughout all body positions but particularly during standing. Indeed, SOL appears to be the only muscle undergoing inhibitory effects during standing. This response is in agreement with the early work of Preston et al. [31] and subsequent work of Cowan et al. [32], implying that the corticospinal excitatory drive to SOL, the drive responsible for the twitch seen in active muscles, arrives at the SOL motoneurons later than the afferent sensory inputs, possibly due to a larger amount of synapses that have to be crossed. The excitatory second responses seen particularly in RF may also be explained by pre-synaptic activation of cortico-motoneural projections, which are known to greatly facilitate spinal excitability, and may be less significant or absent in SOL. Indeed, the ratio of motorcortical to Ia afferent inputs is thought to be related to the functional demands of discrete motor pools [33]. This variance in motor pool input ratios is most clearly observed when TMS is applied to evoke TA and SOL [34]. While PRMR1 responses in standing were less than supine and supine 90-90, primarily due to Ia afferent pre-synaptic inhibition [35], they were not significant and did not agree with previous research [36,37], although these studies only looked at H-reflex. It must also be noted that a second response presence following a particularly large first response does not automatically imply that motor fibres were activated, but rather may imply the rapid recovery of large-amplitude reflexes, as large reflexes are less liable to be altered by a conditioning input [38].

With regards to other muscles, the stronger suppression observed in the TA and SOL compared to RF as seen in Figure 5b may be due to an innate motor control characteristic of the stretch reflexes within RF, suggested in the reduced homonymous pre-synaptic afferent inhibition and or homosynaptic depression after repetitive stimulation in comparison to the more distal muscles of the leg [39]. In addition, as explained earlier, the level of sensory afferent input relative to corticospinal projections varies depending on the functional demands of the muscle [33,34]. Further, SOL and the medial hamstrings have larger diameter Ia afferents compared to RF and *vastus lateralis* [13], which may in part explain the similar recruitment curves of SOL and BF_LH_ seen in Figure 6. The functional and anatomical variance in sensory afferent and corticospinal input synapsing to discrete motor pools at a spinal level may in part explain the differing PRMR1 and PRMR2 responses observed in some positions and muscles. 

The PRMR1 response amplitudes during sitting may be significantly less compared to supine and supine 90-90, but no significant differences were reported in %PAD between sitting and any other body position. Whilst it was not significantly different, motor or mixed-activation may be favoured in sitting, which is in agreement with Binder et al. [20], although they did not statistically compare positions. The post-activation potentiation effect seen in sitting and standing may also be due to a lack of dorsal root engagement due to activation of the back extensors muscles during sitting and standing. 

The biophysical theory put forward by Binder et al. [20] to explain positionally induced changes in evoked responses may be partially discredited as a result of our study, as spinal flexion and extension movements were controlled for through the use of a spinal brace. While the observed differences in RMT and PRMR1 responses in sitting may still be due to biophysical effects, central influences cannot be overlooked. The RMT was statistically lower in supine (74 ± 19 mA) compared to sitting (86 ± 24 mA) positions, yet it was even lower in supine 90-90 (70 ± 23 mA), even though they are anatomically the same position. The reasons for this may still be biophysical, with compression of the sciatic nerve via the chair during sitting. The anatomy of the piriformis muscle may also play a significant role in the compression of the sciatic nerve during sitting as the nerve exits the pelvis just below the belly of piriformis [40]. Multiple variations in this anatomy also exist; in some, the nerve may separate proximally to the muscle, and in others, the nerve will dissect the piriformis [41]. Furthermore, the nerve has two main divisions, the tibial and peroneal divisions [42], which are also covered superficially by the upper segment of tensor fascia lata, and then the gluteus maximus muscle. This musculature combined with compression against the ischial tuberosity during sitting [43] may have influenced the RMT and PRMR1 response amplitudes, and subsequently %PAD, potentially explaining the significantly higher RMTs compared to the supine 90-90 position. 

### Limitations and Future Research

The data collected and analysed during this study were taken from a healthy neurologically intact cohort; therefore these results cannot be considered directly applicable to those with an SCI, the main beneficiaries of tSCS treatment. The sample was also relatively small, with a sample size for body position comparison of just 11 individuals, although this is a greater number compared to other research in this area [20,26]. Considering PRMR2 responses can be seen in body positions at 10 ma (Figure 6), future research could begin stimulation at 1 ma and continue in 1 ma increments. Further, cathode (T11/12 intervertebral space) and anode (either side of the umbilicus) placement was consistently maintained throughout testing, yet the broad anatomical variation along the rosto–caudal axis in the enlargement of the lumbosacral region has been shown to exist, resulting in varied responses between participants [13,44]. This may have been avoided if a familiarisation session had been used to locate optimal cathode placement, an area of research also requiring further investigation. Future research could also investigate PRMR1 response amplitudes and %PAD close to RMT intensity, as this is typically the starting intensity of the therapeutic tSCS dosage.

## 5. Conclusions

There is a lack of consideration, in the form of minimal research conducted in the area, regarding body positional effects on PRMR-evoked responses during tSCS, which has led to incomparable results across tSCS research. This study has hopefully reduced this to an extent. Although the above limitations hinder the magnitude of the observed results, this study provides further evidence that body position significantly influences PRMR RMTs and evoked responses, and will likely do so during activities like standing from a seated position. Sitting has higher RMT values compared to supine, supine 90-90 and standing body positions. Supine 90-90 has the lowest RMT, which may indicate it as the “optimal” starting body position for tSCS therapeutic intervention in those with a SCI. Furthermore, supine and supine 90-90 body positions favour the stimulation of sensory afferents compared to sitting and standing, while the standing body position caused greater activation or co-activation of efferent fibres. Considering this is only the third study to investigate body positional effects on the PRMR responses and the first to statistically investigate sitting, more research is required to better understand the influence body position may have.

## Data Availability

The specific MATLAB scripts used in data processing (Section 2.6) are available upon request.
